# Annual International Geographic Medicine Congress Meetings in Shiraz, Iran: Publication Rates during 1999-2006

**Published:** 2009-03

**Authors:** Forouz Nader, Kazem Mousavizadeh, Pedram Ghafourifar

**Affiliations:** 1 *Department of Community Medicine, School of Medicine, Shiraz University of Medical Sciences, Shiraz, Iran;*; 2 *Department of Basic Sciences and*; 3 *Cellular and Molecular Research Center, Iran University of Medical Sciences, Tehran, Iran;*; 4 *Tri-State Institute of Pharmaceutical Science, Huntington, West Virginia, USA*

**Keywords:** Iran, scientific meetings, publication rate

## Abstract

The present study aimed to examine which portion of abstracts presented between 1999 to 2006 at Annual International Geographic Medicine Congress meetings in Shiraz, Iran, were published during 1999-2006, and to identify factors affecting publication rate of those abstracts. Two hundred fifty abstracts were reviewed and categorized according to the type of presentation, study design, sample size, main findings, source of funding, and statistical significance of the results. Principal investigators of those abstracts were provided with a questionnaire inquiring whether their abstracts lead to full-length publications in peer-reviewed journals indexed under PubMed. One hundred twenty five authors responded to the questionnaire. The publication rate of the meeting presentations was found 27.2%. Statistically significant associations were found between publication rate and certain characteristics of the presentations including type of the study, achieving positive results, and conducting multi–center trial funded by a sponsor. Insufficient fluency in English, insufficient time to prepare the manuscript, and assuming journals are unlikely to accept those studies were most common reasons for not preparing or submitting the manuscripts. The publication rate of research studies presented in this annual scientific meeting in Shiraz, Iran, is lower than many similar meetings in other countries.

## INTRODUCTION

Factors influencing publication of biomedical studies have been the subject of increasing attentions. It has been shown that demographic distribution of authors, type of study, sample size, source of funding, and the results obtained influence publication of medical research (1-9). Significant portion of studies presented at scientific meetings do not lead to full-length publications in biomedical journals indexed under PubMed. Many investigators present the results of their studies in forms of abstracts in the meetings. However, those abstracts are not available to the general scientific community. Additionally, abstracts often do not undergo rigorous peer-review of methods, results, and conclusions as conducted for full-length articles. Consequently, many abstracts presented in scientific meetings do not gain citation (7, 10-17). In early 1979 “the Uniform Requirements for Manuscripts Submitted to Biomedical Journals” explicitly advised authors to “try to avoid using abstracts as references” (15-19). However, many journals do not draw a coherent policy regarding citation of the conference abstracts.

The aim of the present study was to investigate the characteristics of abstracts presented at the Annual International Geographic Medicine Congress meetings during 1999-2001, identify which portion of those abstracts were published during 1999-2006, and delineate factors hindering publication of certain abstracts as full-length papers. Annual Geographic Medicine Congress is one of the leading scientific meetings held since 1984 in Shiraz Medical School, Shiraz, Iran, hosting presentation on various biomedical subjects.

## METHODS

### Selection and description of participants

Multi-stage random sampling was used to select 250 abstracts from all 615 abstracts submitted to the Annual Geographic Medicine from 1999 to 2001. Sample size of each year was calculated by using stratified random sampling. Samples were selected by using systematic random sampling from each Congress Booklet.

### Technical information

In March 2006, a cover letter explaining the purpose of our study along with a questionnaire was sent to the principal investigators of the selected abstracts. The questionnaire asked whether the abstract presented during 1991-2001 was published in form of a full-length paper in a journal indexed under PubMed. If the abstract was published, the principal investigator was asked to provide citation to the published study. For those studied did not lead to a publication, it was asked whether the study was submitted for publication. If the study was not submitted for publication, the principal investigator was asked to provide reason(s) for not submitting the manuscript.

In January 2007, a PubMed search was conducted using authors’ names or combination of authors’ names and the keywords provided in the abstracts. A study was considered published if the article was found under PubMed or the principal investigator stated that the study was accepted for publication.

Each abstract was randomly assigned to 2 investigators for classification of the study characteristics. These investigators extracted the study characteristics by carefully studying each abstract, identifying study features, and collecting the data for statistics. Items extracted included number of centers where the research was conducted, *i.e.*, uni-central vs. multi-central, design of the study, source of funding, sample size, main findings, obtaining positive or negative results, and statistical significance of the results. The investigators were blinded with respect to authors and whether the abstracts were published in a PubMed-indexed journal. A third investigator resolved disagreements regarding the study characteristics.

### Definitions

**Study:** A study was defined as one in which one or more variables were controlled to monitor the effect on a process or outcome. A study was considered observational when a process or disease was observed without intend to alter the process or disease during the study.

**Publication:** A publication was considered an article published or accepted for publication in a journal indexed under PubMed. Book chapters and published meeting abstracts or proceedings were not considered publication.

**Type of study:** Studies were categorized into two large categories of Experimental vs. Observational.

### Statistics

Data extracted from the abstracts and questionnaires were analyzed by using SPSS version 10. Association between study characteristics and publication was estimated by using Chi square and Fissure Exact tests as described (20). Differences were considered significant if the main outcome attained a P value of less than 0.05, and non-significant if the P value was equal or greater than 0.05.

Certain study characteristics were evaluated in a backward logistic regression to determine which factors influenced publication rates. In the multivariate analysis, candidate variables entered into the model using a liberal P<0.15 criterion. Criterion variable for logistic regression was set publication in a journal indexed under PubMed.

## RESULTS

Of 250 selected abstracts, 125 principal investigators replied to our letters, providing an overall respondent rate of 50%. No difference was found between studies for which the principal investigators replied to the questionnaire and those did not, in terms of the number of studies accepted per investigator for presentation in the meeting, time of the meeting, affiliation of investigators, or type of the study.

### Pursuit of publication

Characteristics of 125 abstracts are summarized in Table 1 that shows the percentage of research studies published within 7 years after the abstracts were presented. Associations were found between publication rate and certain characteristics of researches such as type of presentation, type of study, achieving positive results, conducting in form of a multi-central trial, and funding by a sponsor. Publication rate of abstracts presented in oral form was greater than those presented as poster (48.3% vs. 20.9%, P=0.02). Publication rate of observational studies was lower than experimental studies (20.5% vs. 40.42%, P=0.03). Obtaining positive results that were reported in 76.4% of presentation affected the publication rate (35.41% vs. 3.44%, P=0.0001). Abstracts representing researches financially supported by sponsors were published more frequently than those without financial sponsors (54.34% vs. 12.65%, P=0.001). Multi-center studies were found more likely to be published compared to those performed uni–centric (55.55% vs. 25.23%, P=0.01). Publication rates did not show significant association with sample sizes (P=0.616; Table 1).

**Table 1:** Characteristics of the researches

	Number of abstracts (%)	Number of abstracts published as full-length paper (%)

Total	125 (100)	34 (27.2)
Type of presentation		
Oral	30 (24)	14 (48.3)
Poster	95 (76)	20 (20.9)
Type of study		
Observational	78 (62.4)	16 (20.5)
Experimental	47 (37.6)	19 (40.42)
Sample size		
≤100	81 (64.5)	23 (28.39)
Between 101 to 500	33 (26.6)	8 (24.24)
≥ 501	11 (8.9)	3 (27.4)
Results		
Positive	96 (76.4)	34 (35.41)
Null or negative	29 (23.6)	1 (3.44)
Number of center		
Multi-Center	18 (14.4)	10 (55.55)
Uni-Center	107 (85.6)	27 (25.23)
Financial sponsor		
Specified	46 (36.8)	25 (54.34)
Unspecified	79 (63.2)	10 (12.65)

Multivariate logistic regression analysis (Table 2) revealed that factors independently associated with increased likelihood of full-length publications were: obtaining positive results, being funded by financial sponsors, and presenting the research in form of oral presentations.

**Table 2:** Factors associated with the rate of publication in Multivariable Model

Variables	Hazard Ratio (95% Confidence Interval)	P Value

Type of result		
Positive	1.5 (1.2-1.5)	0.02
Null or negative	1	
Financial sponsorship		
Specified	1.49 (1.05-2.12)	0.04
Unspecified	1	
Type of presentation		
Oral	1.4 (1.06-1.9)	0.02
Poster	1	
Sample size		
Above median	1.2 (0.9-1.7)	0.24
Below median	1	
Number of Center		
Multi-center	0.86 (0.60-1.23)	0.4
Uni-center	1	
Type of study		
Experimental	0.79 (0.57-1.10)	0.16
Observational	1	

### Reasons for failure to publish

Among 91 abstracts that did not lead to a full-length publication, 92.3% were never submitted and only 7.7% were submitted and not accepted for publication. The most common reasons for not publishing full-length publications were lack of sufficient time to prepare the manuscript, insufficient fluency in English, and the assumption that journals will not accept the study for publication (Table 3).

**Table 3:** Reasons for lack of publication of abstracts presented in Annual International Geographic Medicine Congress

Reasons	Number of respondents (%)

Journals unlikely to accept	26 (28.57)
Results lack sufficient importance	7 (7.7)
Manuscript in preparation/under review	17 (18.68)
Lack of time to prepare the manuscript	26 (28.57)
Insufficient fluency in English	18 (19.78)
Article submitted but not accepted for publication	7 (7.7)
Other reasons	5 (5.5)

## DISCUSSION

Subsequent publication as full-length articles in peer-reviewed journals of abstracts presented in scientific meetings could be a measure of quality. It has been shown that many studies do not lead to a full-length peer-reviewed publication (8-12). The present study is one of the few investigations aimed to reveal the publication rate of researches in Iran. Our findings show that only 27.2% of meeting abstracts were subsequently published in 7 years as full-length papers in journals indexed under PubMed. Our finding corroborates with previous studies of medical meetings reporting publication rates from 25 to 60% (21). Evaluating the publication rate for such long duration as of 7 years after presentation allows accreting whether a manuscript was published. Most unpublished studies were never submitted to be considered for publication in a peer-reviewed journal.

Studies have suggested several factors contribute to subsequent publication of an abstract in form of a full-length paper. Those include achieving positive results, utilizing large sample size, study design comprising of randomized controlled trial, oral presentation in a conference or other scientific gatherings, and sponsorship by external funds (1, 13, 22-30). Our study found that certain abstracts are more likely to be published as full-length articles. Abstracts presented in form of oral presentations were published more than those presented as posters. Experimental studies were published more than observational studies, and studies resulted in novel positive findings showed publication rate higher than those found null or negative results. Likewise, publication rate of studies conducted multi–centrally was higher than those conducted uni-centrally. Finally, researches financially supported by a sponsor showed publication rate higher than those with no specified sponsor. Multiple logistic regression analysis confirmed there is no association between publication rate and factors including the sample size and the type of study. This finding can be described by several factors. Those factors include categorizing the studies into Observational and Experimental, and that most researches with large sample size were descriptive that do not warrant publication in many international journals. Additionally, case series studies that are categorized Observational are more likely to be considered for publication in international journals. In agreement with our findings, a previous study revealed that sample size and study type did not significantly influence the publication rate (21).

The exact reasons certain studies do not lead to publication are not fully understood. Consistent with our findings, others have shown that meeting abstracts that showed benefits of a drug or medical device had much greater publication rate as full-length papers than those showed negative results or no benefits (31, 32).

Likewise, our findings support prior reports suggesting that most unpublished researches were never submitted to a journal (18, 24, 30-38). While abstracts are published in conference proceedings, lack of rigorous peer-review does not allow conference proceedings take place of full-length scientific publications. Additionally, conference proceedings are not available at large to the scientific and medical community. Our findings indicate that most common reasons for not submitting the abstract in form of a full-length manuscript are insufficient time to prepare the manuscript and the assumption that the journals are unlikely to accept the manuscript. These findings support findings of other studies (23, 27, 30, 33-37) and suggest similarities between the researchers studied under our study and others.

It has been shown that English proficiency is strongly associated with successful publication in high-ranking medical journals (38-42). Our findings show insufficient fluency in English might be a major (19.75%) reason underlying lack of submission of researches for publication. Insufficient fluency of authors in English along with lack of sufficient PubMed-indexed Persian journals can be considered major drawbacks for Iranian scientists to publish their findings.

Each study bears limitations. In our study, only 50% of principal investigators replied to questionnaires and only PubMed-indexed journals were considered. Previous studies reported response rates from 54 to 62% (21). Moreover, the present study evaluated the study design, main findings, and statistical significance of results in the abstracts, while the interpretations of the results were not evaluated. Finally, the present study considered publications only up to 7 years after the meeting. Although more than 90% of researches are published within 4 years after their presentations in scientific meetings (13), it is possible that a small portion of abstracts publish afterwards. Our study did not aim to evaluate whether several abstracts were consolidated to create one full-length manuscript for publication. However, it should be considered that meeting abstracts are limited in space and authors commonly combine several meeting abstracts to form one full-length manuscript to submit for publication.

The present study offers critical implications. While Iranian clinicians and scientists wish to publish their clinical and research findings, lack of publication of numerous researches performed in Iran limits dissemination of the information obtained and accessibility to that information. Analysis of the frequency and typology of scientific papers from developing countries shows that a vast majority of those papers are published in journals with unknown Impact Factor, such as those published only at national level. This leads to a serious under-representation of studies conducted in developing world in high-impact publications. Consequently, developing countries do not attain the opportunity to contribute to the advancement of biomedical sciences. Additionally, lack of publication of scientific findings adversely affects decision making in medical practice (27, 30-37). We found that 63% of the abstracts were subsequently published in journals that are not indexed under PubMed, including Persian medical journals. Although some of those journals may perform peer-review of the manuscript less rigorous than international high-ranking journals, information published in Persian journals can be used in local health and medical decision-making. Our findings indicate the need for highly qualified PubMed-indexed Persian medical journals.

Several approaches can be taken to improve the publication rate. For example, investigators, sponsors, as well as institutional committees should ensure the quality of study design, data collection, and interpretations are comparable to studies published in high-ranking journals. Researchers should be encouraged to publish results of their studies and incentives should be offered to those publish full-length papers in peer-reviewed high impact journals. Institutions in developing countries should foster collaborative efforts between their scientists and clinicians with scientific and academic medical centers of developed countries with extensive publication record. Such effort will assist researchers in developing countries to obtain additional skills in designing the study and preparing the results for publication.
